# Socio-Economic and Cultural Determinants of Human African Trypanosomiasis at the Kenya – Uganda Transboundary

**DOI:** 10.1371/journal.pntd.0002186

**Published:** 2013-04-25

**Authors:** Jane Jemeli Rutto, Odipo Osano, Elias Gitonga Thuranira, Richard Kiptum Kurgat, Victor Agab Omondi Odenyo

**Affiliations:** 1 Kenya Agricultural Research Institute, Trypanosomiasis Research Centre, Kikuyu, Kenya; 2 Chepkoilel University College, Moi University, School of Environmental Studies, Eldoret, Kenya; 3 Kenya Agricultural Research Institute, National Agricultural Research Laboratories, Nairobi, Kenya; Foundation for Innovative New Diagnostics (FIND), Switzerland

## Abstract

**Background:**

Kenya and Uganda have reported different Human African Trypanosomiasis incidences in the past more than three decades, with the latter recording more cases. This cross-sectional study assessed the demographic characteristics, tsetse and trypanosomiasis control practices, socio-economic and cultural risk factors influencing *Trypanosoma brucei rhodesiense* (*T.b.r.*) infection in Teso and Busia Districts, Western Kenya and Tororo and Busia Districts, Southeast Uganda. A conceptual framework was postulated to explain interactions of various socio-economic, cultural and tsetse control factors that predispose individuals and populations to HAT.

**Methods:**

A cross-sectional household survey was conducted between April and October 2008. Four administrative districts reporting *T.b.r* and lying adjacent to each other at the international boundary of Kenya and Uganda were purposely selected. Household data collection was carried out in two villages that had experienced HAT and one other village that had no reported HAT case from 1977 to 2008 in each district. A structured questionnaire was administered to 384 randomly selected household heads or their representatives in each country. The percent of respondents giving a specific answer was reported. Secondary data was also obtained on socio-economic and political issues in both countries.

**Results:**

Inadequate knowledge on the disease cycle and intervention measures contributed considerable barriers to HAT, and more so in Uganda than in Kenya. Gender-associated socio-cultural practices greatly predisposed individuals to HAT. Pesticides-based crop husbandry in the 1970's reportedly reduced vector population while vegetation of coffee and banana's and livestock husbandry directly increased occurrence of HAT. Livestock husbandry practices in the villages were strong predictors of HAT incidence. The residents in Kenya (6.7%) applied chemoprophylaxis and chemotherapeutic controls against trypanosomiasis to a larger extent than Uganda (2.1%).

**Conclusion:**

Knowledge on tsetse and its control methods, culture, farming practice, demographic and socio-economic variables explained occurrence of HAT better than landscape features.

## Author Summary

The prevention and control of Human African Trypanosomiasis depends on application of knowledge and appropriate technologies to modify tsetse ecology by community members and relevant stakeholders. In our study Ugandan districts reported more *T.b.r.* than the neighboring districts in Kenya in the past 30–50 years. Historical, political, social and economic factors have influenced the ecology of HAT vectors and its prevalence in both Western Kenya and Southeast Uganda. The villagers' participation in different conventional and traditional methods of tsetse control influenced HAT occurrence and distribution in the study area. Cattle husbandry practices and marketing may affect the spread of HAT disease as reported in Western Kenya and Southeast Uganda. Land use had immense influence on the occurrence of the tsetse flies and wildlife carriers of trypanosomiasis. The modification of vegetation cover through bush clearing reduced habitats suitability to tsetse flies. The respondents' occupation and gender roles played a role in influencing the interaction between humans and tsetse flies. Human African trypanosomiasis could be controlled effectively by modifying both physical and socio-political factors that affect the interaction of tsetse flies and human beings.

## Introduction

Human African Trypanosomiasis (HAT) or Sleeping sickness caused by *Trypanosma brucei* protozoa and transmitted by tsetse fly (*Glossina* spp) vector is found only in Sub-Saharan Africa, within 36 countries lying between latitudes 14° North and to 29° South. An annual incidence ranging from 50,000 and 70,000 of HAT was been reported for the past 50 years until 2009 when new cases per annum dropped to below 10,000 [1] which further reduced to 6,743 cases in 2011 [2]. This decline was achieved through concerted campaigns led by World Health Organization (WHO), and many nongovernmental organizations [3].

HAT is caused by *Trypanosoma brucei rhodesiense* (*T.b.r)* and *Trypanosma brucei gambiense* (*T.b.g.)*, with the former being commonly found in Eastern and Southern Africa, while the latter is in Central and West Africa. *T.b.r* causes acute illness with untreated cases dying within three to five months of infection while *T.b.g*. causes chronic infection lasting several years [4]. *T.b.r* form of HAT is endemic in our study area [5], [6].

The main biophysical determinants of transmission are the presence of the tsetse fly and mammalian reservoir hosts. In addition, social determinants of the health (SDH) including income, education, occupation, gender, race/ethnicity, culture and other factors may have a potential to influence the outcome of HAT [7], [8]. HAT remains an endemic and a neglected tropical diseases (NTDs) in poor Sub-Saharan regions where prevailing political, social, cultural, economic and physical environment do not allow for formulation of appropriate interventions strategies [9] for vector and disease control.

Uganda has reported higher cases of *T.b.r* form of HAT in the past over 3 decades compared to Kenya. It has been reported that the nature and duration of the contact which could either be personal (intimate) or impersonal (casual) determines the intensity of transmission to humans [10], [11]. Thus understanding land use and land cover characteristics, tsetse and their infection rates with *T.b.r.*, animal reservoirs, lifestyle activities such as livestock keeping practices, cattle numbers, livestock marketing, human activities, grazing and watering points which influenced incidence of HAT in western Kenya and southeastern Uganda was necessary. Other concomitant factors such cultural practices also contributed to HAT incidence.

Here, we describe interactions between socio-economics, culture, tsetse control methods and environmental issues and *T.b.r*. infection at Kenya and Uganda transboundary. We also examined the application of conventional and non-conventional tsetse control strategies over time and existing landscape features at homesteads and how such transformations influenced HAT occurrence at the transboundary of Kenya and Uganda. The study hypothesis was that land use, economic, socio-cultural, livestock keeping practices, livestock marketing, tsetse and trypanosomiasis control practices influenced HAT dynamics in affected villages of Western Kenya and Southeast Uganda.

## Materials and Methods

### Study Area

The study site was located in Kenya (Ke) and Uganda (Ug) transboundary in an area comprising four districts namely Tororo (Ug), Busia (Ug), Busia (Ke) and Teso (Ke). Busia (Ke) and Teso (Ke) Districts (within Busia County) are located in Western Kenya between latitude 0° and 0°45′ North and longitude 33°54′ east and 34°25′east [12], [13]. Tororo (Ug) and Busia (Ug) Districts are located in Southeastern Uganda and lie between latitude 0° and 0°45′ North and longitude 34° and 34°15′ East [14]–[17]. The tsetse vectors present are *Glossina pallidipes* and *Glossina fuscipes fuscipes*.

### Ethical Statement

The study protocol and the use of informed oral consent were approved by the Board of Graduate Studies, Moi University, Kenya. Also permission was sought from Districts administration through the village chiefs and village elders before the interviews in Western Kenya and Southeast Uganda. Interviews using pre-tested structured questionnaires were conducted systematically to randomly selected household heads or their representative which was either a spouse or older child of above 30 years. Most of the respondents were illiterate or semi-literate and could not read or write, therefore oral consent was sought from all the participants for uniformity. After obtaining verbal informed oral consent the research assistant interviewed the respondent and if the respondent declined another household was sought. All interviewed persons provided informed oral consent witnessed by an administrative head (the chief or village elder) who also acted as the village guide. The verbal oral consent was documented by the respondents providing an affirmative tick mark in the provided acceptance space in the questionnaires and each sampled household was geo-referenced.

### Study Design

**Figure 1:**
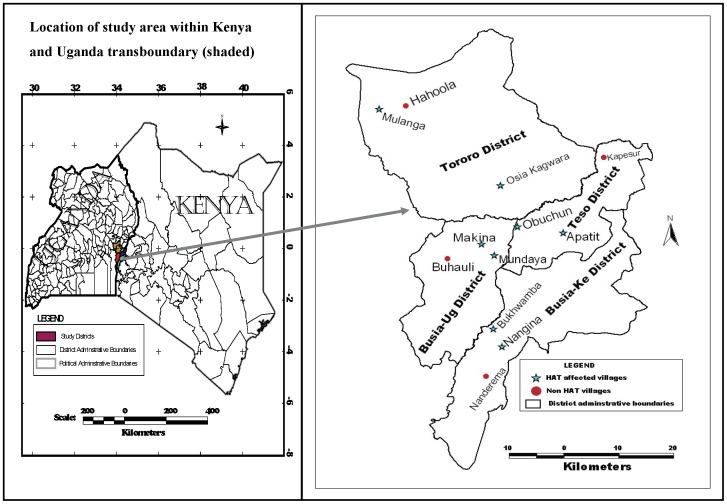
Selected study villages in Kenya and Uganda transboundary.

The study was a cross-sectional household survey conducted between April to October 2008. The four districts were purposely selected on the basis of them being foci for *T.b.r.* type of HAT in both countries and that they lie adjacent to each other at the international boundary of Kenya and Uganda. Then sub-locations/sub-counties from each district with relatively higher reports of HAT cases were purposively selected. The sampling unit was the village; therefore all the villages within the sub-locations and sub-counties constituted the sampling frame. Household data collection was conducted in 12 villages comprising 3 villages from each of the 4 study districts namely; Teso (Ke), Busia (Ke), Tororo (Ug), and Busia (Ug). In both countries 2 of the villages in each district had experienced HAT (HAT affected villages) and the other one village had no reported HAT case (unaffected villages) from 1977 to 2008 (fig. 1). The study stations comprised centers of the village which was either school, church or trading center representing the dispersal point from where systematic sampling was used to identify the households. In order to conduct a detailed analysis of risk factors at the household level, 8 villages that had reported high number of HAT cases ranging from 3 to above 100 HAT cases from 1977 to 2008 were selected.

The hospital records of HAT affected villages in both Kenyan and Ugandan from 1977 to 2008 kept and stored by the Trypanosomiasis Research Centre (TRC) in Kikuyu, Kenya, and the National Livestock Health Research Institute (NALIRI) in Tororo, Uganda were used. All the HAT affected villages were mapped and control villages (unaffected villages) points were marked randomly in locations within the study area. The coordinates of the marked point was uploaded in a GPS machine and the village was traced to its actual position on the actual ground. The name of the village was obtained through government administrative officers and the local residents. The authors acknowledge that some villages may not have reported HAT cases due to misdiagnosis, therefore not referred to HAT referral hospitals in both countries [18]. The study stations comprised centers of the village which was either school, church or trading center representing the dispersal point from where systematic sampling was used to identify the households.

### Data Collection Techniques

Interviews were conducted randomly to selected household heads or their representatives. The questionnaire was first designed in English and then translated and pre-tested in local languages well understood by the participants. The languages of the respondents were Teso, Luhyas, Luo, and Samia in Western Kenya and Teso, Adhola, Nyole, Mugwe, Musoga and Samia in Southeast Uganda. Ten enumerators were recruited from the various tribes in the study districts in each country. Every third household was sampled. In each country 384 households were sampled. This was a retrospective study and elderly individuals above 30 years were interviewed. If the household head or their representative who was either a spouse or child was less than 30 years, the household was skipped and the immediate third household was sampled. A child in this paper refers to a boy or daughter who were not married and living in their parents homestead or did not have their own households at the time of the interview. The respondents were given information about the objectives of the study using the local language of the participants' preference. A minimum of 63 respondents from each of the selected 6 villages in each country were interviewed. The sample size formula was statistically determined using Rasosoft program [19].

The sample size n and margin of error E were given by:
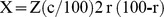


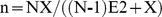


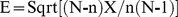



Where N was the population size, r is the fraction of sample collected, and Z(c/100) was the critical value for the confidence level c in this study 95% confidence level was used therefore, the minimum sample size required was 384 from each country. The official human population in Western Kenya study districts were 552,099 in 1999 [12], [13], [20] while in Southeast Uganda it was estimated at 887,345 in 2002 population census [16], [17]. The geographic coordinates of all homesteads in these villages were mapped using a hand-held Garmin Global Positioning System (GPS). The questions focused on various sub-themes like the socio-demographic characteristic of the respondent, gender, age, tribe, knowledge and perception on tsetse and trypanosomiasis, historical tsetse and trypanosomiasis control methods, livestock keeping practices, land size, land use patterns, occupation, human dwellings, cultural and behavioral practices, other family practices related to HAT transmission and perceived effectiveness of available control programs. The questionnaires were prepared in English and verbally translated into local language during the interview time. The percent of respondents giving a specific answer was reported. Secondary data through literature was also obtained on socio-economic and political issues in both countries. The authors acknowledge that in many retrospective studies, the quality of the information obtained can be affected by recall bias. In the present study, to minimize the inaccuracy resulting from errors in recall, information obtained directly from the respondents was verified by interviewing their spouses and elder children above 30 years.

### Conceptual Framework

**Figure 2:**
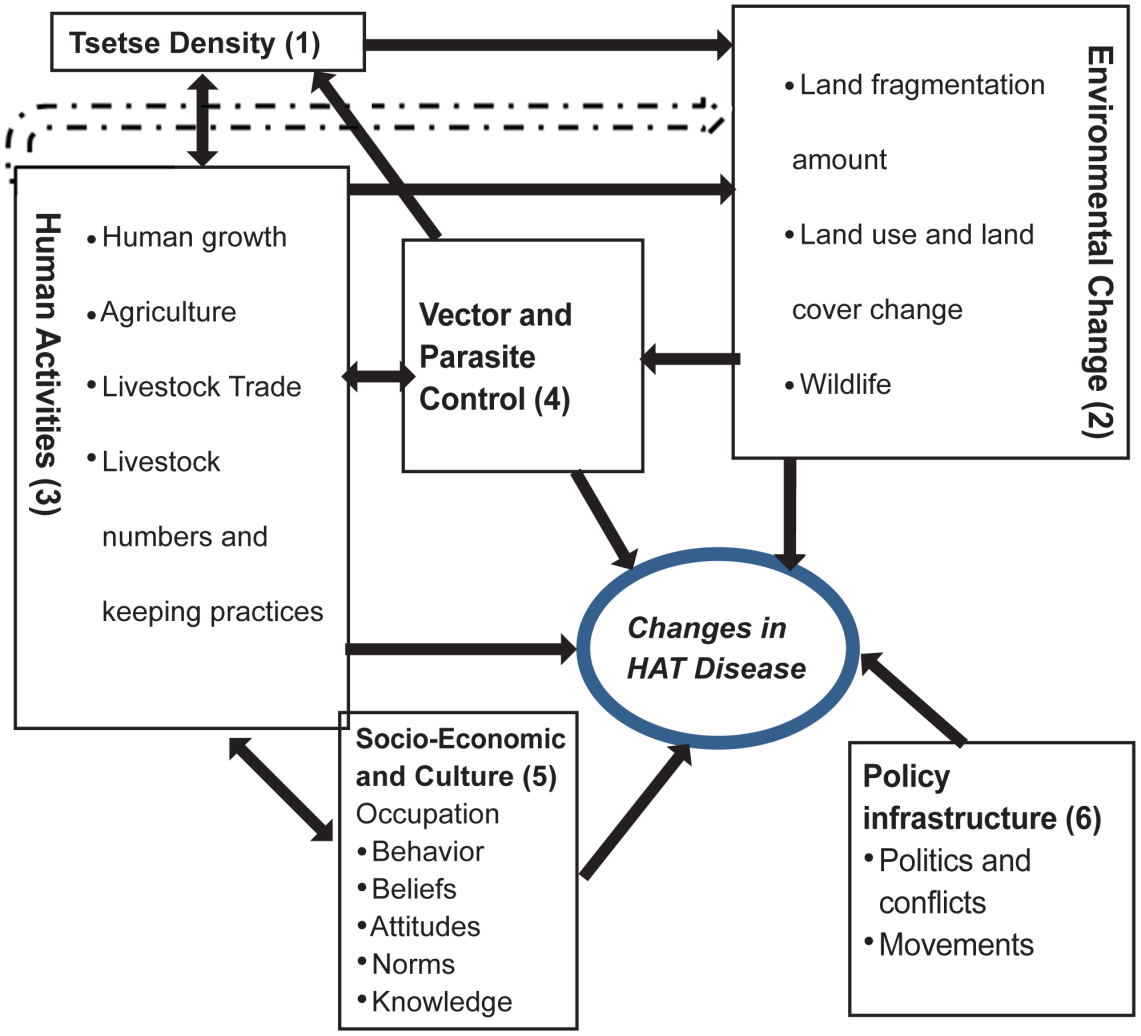
A conceptual framework for tsetse and trypanosomiasis control in Kenya and Uganda transboundary.

A conceptual framework was postulated to explain interactions of various socio-economic, cultural, biological and physical factors that predispose individuals and populations to HAT (fig. 2) at Kenya and Uganda transboundary.

#### Tsetse density

High tsetse numbers enhances possibilities of human/fly contact and possible HAT transmission. Land fragmentation lowers the tsetse populations and generally natural woody vegetation enhances the numbers of tsetse flies [21]–[24]. The location of the human dwelling within a forested area or conducive tsetse habitats increases HAT risks due to human-vector interactions. For HAT to be transmitted, human-fly contact needs to be regular and intensive, and the density of insect vector population determines the potential per capita risk burden on host.

#### Environmental

Tsetse flies distribution and abundance are influenced by existing land cover and land use (LULC) which are defined by climate and anthropogenic activities. The relationship between tsetse populations and Normalised Vegetation Index (NDVI) has been well-documented [25], [26]. Tsetse flies' suitable habitats are generally woody vegetation [21], [23], [26]. Land use changes towards more cropland reduces tsetse numbers [27]. In the study area wildlife reservoirs inhabits thick vegetation and swamps and generally share their watering points with livestock and humans. LULC determines the abundance and wildlife species, whereby these wildlife acts as tsetse flies hosts.

#### Human activities

Environmental changes due to anthropogenic activities have led to modifications of tsetse habitat availability thus leading to reduction in vector abundance with a predicted possibility of tsetse extinction [21], [24]. Land use has influence on the tsetse population and is related to the population who deplete the wooded shrubland, natural forest wetland and other woody vegetation to create room for settlement, agriculture or use them for building and construction. Pesticides – based agriculture also compromises the tsetse habitats [28] as opposed to culture of coffee, and banana plantations which are known to promote breeding of the vector in addition to reducing distance to human hosts [29], [30].

Livestock particularly cattle and pigs act as reservoirs of human infective *T.b.r.* and as host for the tsetse fly [1], [31]–[35]. Chemoprophylaxis and chemotherapy on livestock and human reduce circulating parasites and routine topical insecticide application has knock-down effect on tsetse flies. Livestock markets may also increase HAT prevalence as movement of untreated cattle bring the disease to new areas [36].

#### Vector and parasite control

HAT can be prevented through integrated vector control using bush clearing (tsetse habitat removal), wildlife elimination, insecticide application, bush fires, traps and targets, and chemoprophylaxis/therapy, however, knowledge of these methods is an important determinant for their application.

#### Socio-economic and culture

Socio-economic activities such as herding, fishing, and crop farming may predispose individuals to HAT. Gender roles within the study communities are often structured whereby each gender has assigned duties like livestock herding, fishing, crop farming, and collection of firewood. A good knowledge and understanding of tsetse dynamics, its habitats, routes of infection parasite life cycle, and available control methods forms an important background in decision making processes within the community and government agencies for successful tsetse and trypanosomiasis control. Illiteracy and inadequate formal education hampers information flow and understanding on tsetse and disease dynamics. Culture influences human activities and how they perceive disease and their surroundings. Culture determines learned social behavior, occupation and knowledge which are often gender specific and could alter interactions between hosts, parasites and vectors thus impacting on vector-borne diseases [37]. In particular livestock herding, fishing, crop farming, and collection of firewood [10], [37]–[39] have been implicated to be major factors that predispose individuals to HAT of which some of this activities are gender specific. Mitigation measures applied by exposed communities include wearing protective clothing, and staying indoors during certain hours. The study concentrated on the kind of behaviors that were related to HAT outcome.

#### Policy and political infrastructure

Conducive political environment is a precursor for sound policy formulation and action directed at the control of tsetse flies and HAT. Disparities in the political histories of Uganda and Kenya were put into the perspective in this paper. Movement of people and livestock during the political unrest to HAT endemic foci could possibly contribute to the increased HAT incidence in Uganda than Kenya transboundary.

### Management and Analysis of Data

Both qualitative and quantitative data collection methods were used in this largely descriptive study. Historical human activities aimed directly or indirectly at tsetse control were collected from the households in Uganda and Kenya transboundary. The data were entered into Excel 2003 and transferred to Statistical Package for Social Science (SPSS) version 14.0 version for Windows for further statistical analyses. The characteristics of the households in the two countries comprising Kenya and Uganda were described using tables of frequencies. Demographic characteristics such as gender, age, tribe, level of education knowledge on tsetse and trypanosomiasis and their control methods, livestock keeping practices, land size, land use patterns and occupation were compared statistically. Comparisons of the two countries and correlation among the variables were analyzed using Pearson's Chi-squared tests. The relationship between land use patterns in HAT affected and unaffected villages on the occurrence of diseases was investigated through Analysis of variance (ANOVA) [40] tests. Estimation of relative HAT risk was made at 95% confidence level (CL) and set at a significance level of 5% to correlate the strength of HAT and the variable of interest in the two countries, HAT affected and unaffected villages.

## Results

### Demographic Characteristics

The demographic factors studied included household heads' age, education, ethnicity, gender and occupation. The mean age of the study population in Teso and Busia Districts, Western Kenya was 59.0, SE ±2.7 years while in Busia and Tororo Districts, Southeast Uganda it was 52.6, SE±1.9 years. In Teso and Busia Districts, Western Kenya the main ethnic group that were interviewed in the study villages were Teso (50.3%) and Luhya (consisting of Samia, Marachi, Wanyole, and Bukusu sub-tribes) (47.3%). At Busia (Ug) and Tororo (Ug) Districts, Southeast Uganda the inhabitant's tribes of respondents interviewed significantly varied with the major ethnic groups being Samia, Nyole, Adhola and Teso contributing 30.8%, 29.0%, 17.8% and 13.1% respectively. It should be noted that the Teso in the two countries are same ethnicity while the Samia and Nyole of Uganda are similar to the Luhya's in Kenya with regards to ethnicity.

**Figure 3:**
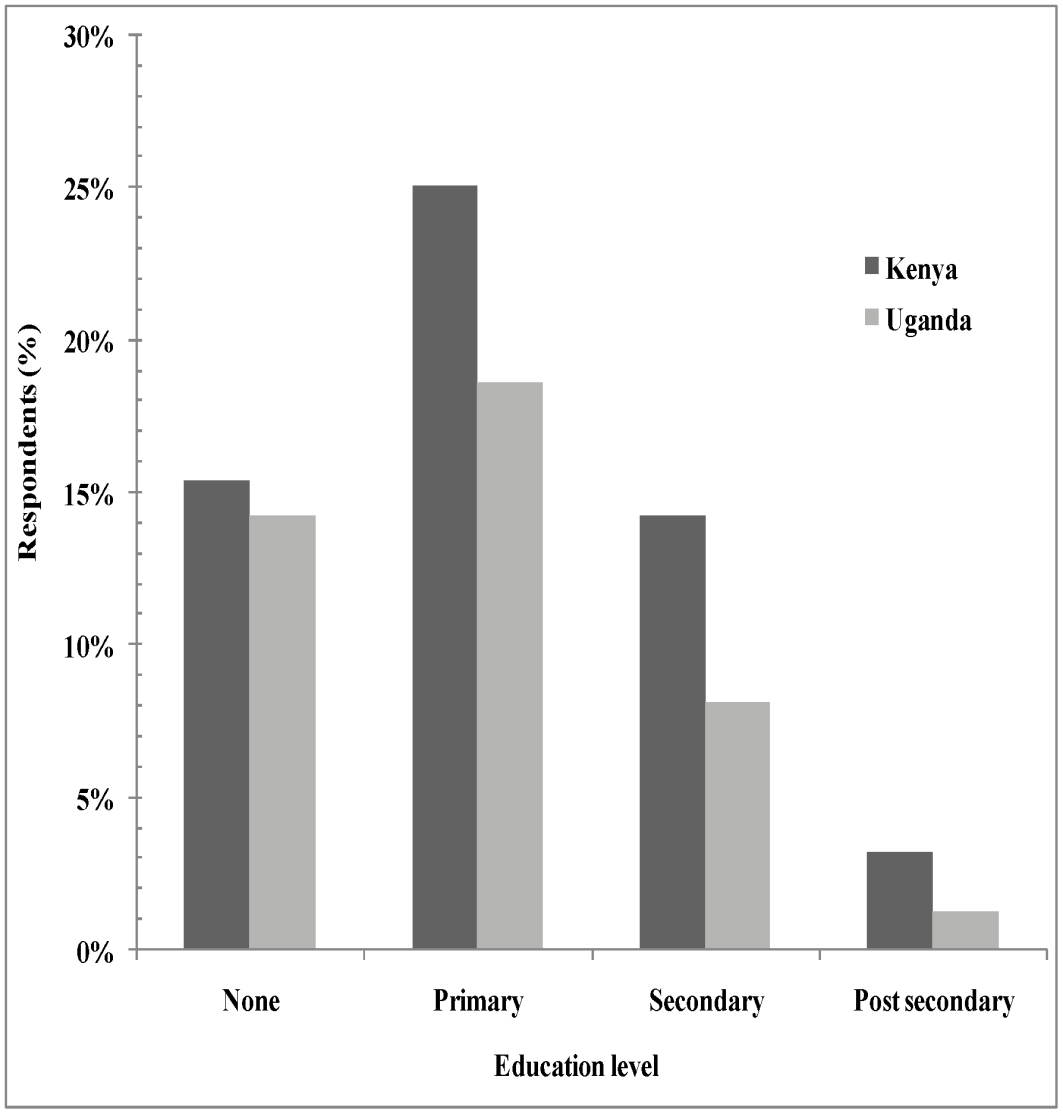
Education levels education in Teso and Busia districts, Western Kenya and Busia and Tororo districts, Southeast Uganda.

The level of primary, secondary, tertiary and no formal education was 25.1%, 15.4%, 3.2% and 14.2%; and 18.6%, 8.1% 1.2% and 14.2%, in Western Kenya and Southeast Uganda, respectively (fig. 3). The respondents' main economic activities were farming, business/trading, employment as a public servant and retired for the old persons who solely depended on pension or relatives at 79.7%, 7.7%, 3.5%, and 5.6% for Kenya and 82.2%, 9.3%, 3.6% and 2.8% for Uganda, respectively.

### Knowledge on Tsetse and Trypanosomiasis

The level of knowledge regarding causes of HAT was high and differences according to the households interviewed in the two countries were minimal. The cause of HAT was well known in both Southeast Uganda and Western Kenya study districts. Household interviews showed that most people were able to associate the presence of tsetse with HAT. Respondents in Teso (Ke) and Busia (Ke) Districts, Western Kenya who knew tsetse flies as the cause of HAT during the survey were 97.9% (n = 376) and the other 2.1% associated it to witchcraft, bushes and mosquitoes. At Busia (Ug) and Tororo (Ug) Districts, Southeast Uganda 82.8% (n = 317) of the respondents knew the cause of HAT was due to tsetse flies, 4.6% that did not know what causes the disease, while 3.7% associated HAT to various reasons like too much rainfall, blood transfusion, bushes and alcohol.

### Gender Risk and Predisposing Socio-Economic and Behavioral Factors

In both countries it was generally reported that the male adults (28.4%) were at higher risk of contracting the disease than female adults (22.9%). The respondents (34.6%) reported that all gender were at risk. The socio-economic activities that contributed to the resident's exposure to HAT vectors in Kenya were herding (51.8%), bathing at the river (14.2%), fishing (10.6%) and other activities had combined contribution of less than 10% (Table 1). In Uganda the important activities that exposed individuals to HAT risk were herding (31.1%), location of homestead in bushy area (12.6%), and bathing in the river (10.3%) (Table 1).

**Table 1:** Perceived HAT risk due to socio-economic activities in Western Kenya and Southeast Uganda.

Activity	Western Kenya (%)	Southeast Uganda (%)
Herding	51.8	31.1
Location of homestead in bushy area	0	12.6
Bathing in the river	14.2	10.3
Fishing	10.6	3.1
Crop farming (along rivers/swamps, bushes and around homesteads)	5.6	7.9
Walking in river/swamp, rocky areas or bushes	6.6	7.7
Fetching water	2.1	4.8
Collection of firewood	1.4	4.6
Washing clothes in the river	1.4	0
Bush clearing	1.4	1.1
Putting on black clothes/walking without shirt	0.7	3.4
Hunting	0.7	2.4
Collection of Building Materials (reeds & poles)	2.8	4.4
Charcoal burning	0.7	3.3
Drinking local brew/eating infected cow	0	2.2
Coffee cultivation	0	1.1
**Total percentage**	**100**	**100**

### Tsetse and Trypanosomiasis Control Practices

The study respondents' historical knowledge on tsetse control methods differed significantly (χ ^2^ = 60.818; df 27, P<0. 001) in the two countries with Western Kenya reporting 62.4% and 37.6% in Southeast Uganda. The selected HAT affected and unaffected villages also differed significantly (χ ^2^ = 25.691; df 9, P<0.001) reporting 65.9% and 34.1% respectively. Sixty one percent of the respondents in Busia (Ke) and Teso (Ke) Districts, Western Kenya knew at least one of the conventional control methods during the interview (Table 2). Of these 34.4%, 21.5% and 0.8% knew traps, bush clearing and ground spraying, respectively for tsetse control. The other known control methods for both tsetse and trypanosomiasis were live bait technology (0.8%) and animal treatment (0.9%). HAT risk was avoided by not bathing in the river and drainage of stagnant water reporting 0.9% and 0.4%, respectively.

**Table 2:** Historical respondents' knowledge on tsetse and trypanosomiasis control methods.

Western Kenya		Southeast Uganda	
Methods	Percent	Traps	Percent
Traps	34.4	Traps	30.6
Bush Clearing	21.5	Bush Clearing	2.5
Ground spray	0.8	Ground spray	0.4
Live bait	0.8	Live bait	0.8
Animal treatment	0.9	Animal treatment	0.4
Medical treatment	0.0	Medical treatment	2.1
Keeping livestock away from homestead	Nil	Keeping livestock away from homestead	0.4
Not bathing in river	0.9	Not bathing in river s	Nil
Avoiding bushy areas	Nil	Avoiding bushy areas	0.4
Drainage of stagnant water	0.4	Drainage of stagnant water	Nil
Putting on white and long sleeved clothes	Nil	Putting on white and long sleeved clothes	0.4
Bush burning	Nil	Bush burning	0.4
Hand catching	Nil	Hand catching	0.4
Don't know	0.7	Don't know	0.8

The recent methods used in the selected villages in Western Kenya were bush clearing, traps and live bait technology contributing 47.4%, and 4.6%, and 3.1%, respectively (Table 3). The live bait technology acted both as a control for tsetse and trypanosomiasis. Also 6.7% of the respondent's used animal treatment to control trypanosomiasis. There was twofold use of traps in HAT affected villages (5%) compared to unaffected villages (2.5%). Similarly bush clearing was twofold in HAT affected villages (51%). However, chemotherapeutic interventions were less varied between the HAT affected villages (5.8%) and unaffected villages (4.2%).

**Table 3:** Comparison of recent methods used for tsetse and trypanosomiasis in Western Kenya and Southeast Uganda.

Western Kenya		Southeast Uganda	
Methods	Percent	Traps	Percent
Traps	4.6	Traps	17.0
Bush clearing	47.4	Bush clearing	13.9
Animal treatment	6.7	Animal treatment	2.1
Live bait technology	3.1	Live bait technology	1.5
Human treatment	Nil	Human treatment	2.1
Traditional medicine	Nil	Traditional medicine	1.0
Avoiding bathing in river	Nil	Avoiding bathing in river	0.5

Thirty nine point two percent of the respondents in Busia (Ug) and Tororo (Ug) Districts, Southeast Uganda knew at least a tsetse control method during the interview (Table 2). Of these 30.6% and 2.5% knew of traps and bush clearing, respectively. Other methods were bush burning, hand catching and ground spraying contributing 0.4%, 0.4%, and 0.4%, respectively. Other historical methods for HAT control included human medical treatment, putting on white and long sleeved clothes and avoidance of bushy areas contributed 2.1%, 0.4%, and 0.4% respectively. The respondents also reported keeping animals away from the homestead (0.4%) and animal treatment (0.4%) contributed to HAT control.

The recent methods practiced in Busia (Ug) and Tororo (Ug) Districts, Southeast Uganda for tsetse and HAT control were traps, bush clearing, avoiding bathing in rivers reporting 17.0%, 13.9% and 0.5% respectively (Table 3). Animal treatment (2.1%) and live bait technology (1.5%) reported as one of the recent methods being used to control HAT. Within the Southeast Uganda study area use of traps in both the HAT affected villages and unaffected villages were reported at 28.4% and 16.2%, respectively. Also bush clearing and livestock treatment within HAT affected villages reported 28.4% and 1.4% respectively while in not affected villages bush clearing and animal treatment reported 5.4% and 4.1%, respectively.

### Socio-Cultural Factors and Possible Interaction with HAT Vectors

Cleansing rituals, followed by circumcision were cited by the respondents as the most important risk factors in both countries. Other activities associated with the disease occurrence were similar in the transboundary and they included exhumation of the dead, appeasing spirits, rain making and marriage where the bride and her age mates wait in the bushes to be offered gifts before being presented to the groom's home (Table 4). The males were considerably more predisposed than the females in all age groups and HAT risk increased with age.

**Table 4:** Percent socio-cultural activities perceived to expose different gender to tsetse bites.

		Gender				
Study area	Cultural activity	Men	Women	Boys	Girls	Total
**Western Kenya**	Cleansing rituals/Ritual bathing	17.0	11.5	9.0	8.0	45.5
	Exhumation of dead/memorials	1.4	4.9	1.4	0.7	8.4
	Marriage (Welcoming women)	4.2	6.8	3.3	0.7	15.0
	Appeasing spirits	3.2	2.1	0.7	0.7	6.7
	Baptism	1.9	0.3	0.0	0.0	2.2
	Rain making	3.0	1.3	0.0	0.0	4.3
	Circumcision	4.0	03	7.6	0.0	11.9
	Traditional healing/medicine	2.5	3.5	0.0	0.0	6.0
	**Total**	**37.2**	**32.7**	**20.0**	**10.1**	**100.0**
**Southeast Uganda**	Cleansing rituals/Ritual bathing	28.0	14.0	8.0	8.0	58.0
	Appeasing spirits	2.1	1.1	1.1	0.0	4.3
	Marriage (Welcoming women)	5.7	6.9	0.0	0.0	12.6
	Circumcision	4.6	1.0	19.5	0.0	25.1
	**Total**	**40.4**	**23.0**	**28.6**	**8.0**	**100.0**

### Land Size and Crops Types Planted by Respondents

The mean land size in Busia (Ke) and Teso (Ke) Districts, Western Kenya was reported to be 2.7 acres. Majority of the respondents in Busia (Ke) and Teso (Ke) Districts in both HAT affected villages (Diseased) and unaffected villages (Not diseased) owned land ranging from 1–5 acres (51.8%) to 6 to 10 (28.7%) acres. The others (19.5%) had < 1 or >10 acres per household. The recent main crops grown in Busia (Ke) and Teso (Ke) Districts, Western Kenya by the respondents were maize (40.8%), cassava (30.3%), finger millet (5.6%) and sorghum (4.9%) (Table 5). Generally acreage of maize and cassava has been increasing, though it experienced some decline in 2000s due to diseases such as cassava mosaic virus attack leading to fewer acres under the crop. According to the respondents, crops such as cotton were grown in 1970s and 1980s but ceased due to lack of markets and unavailability of seeds. The other crops have been almost constant standing crops in the fields within the study area over the study period. Other crops grown by few farmers were soya beans, beans, groundnuts, sesame, yams, pumpkins, sugarcane, vegetables and fruits such as oranges and pineapples. Perennial crops which included bananas and cassava that can act as peri-domestic tsetse habitats were grown by 33.8% of the respondents in the year 2006.

**Table 5:** Respondents historical major crops' percentage cover in Kenya and Uganda.

Study area	Crop	1970s	1980s	1990s	2000	2006
**Western Kenya**	Maize	24.4	31.2	36.2	41.8	40.8
	Cassava	28.2	31.2	34.0	31.2	30.3
	Finger millet	9.2	8.7	8.5	5.7	5.6
	Sorghum	6.9	3.6	4.3	5.7	4.9
	Bananas	2.3	1.4	2.8	3.5	3.5
	Tobacco	1.5	4.3	3.5	2.8	3.5
	Sweet potatoes		2.9	2.9	2.1	2.8
	Cotton	13.7	8.0	-	-	-
	Rice	1.4	1.4	0.7	-	0.7
**Southeast Uganda**	Maize	20.4	16.9	27.2	33.3	37.1
	Cassava	13.0	19.7	21.7	16.7	19.0
	Finger millet	35.2	33.8	29.3	28.3	21.0
	Sweet potatoes	5.6	7.0	2.2	4.0	6.7
	Sorghum	3.7	4.2	4.3	4.0	5.7
	Cotton	14.8	12.7	9.8	7.1	3.8
	Bananas	3.7	4.2	-	2	1.9
	Rice	-	-	-	1.0	1.9

**Figure 4:**
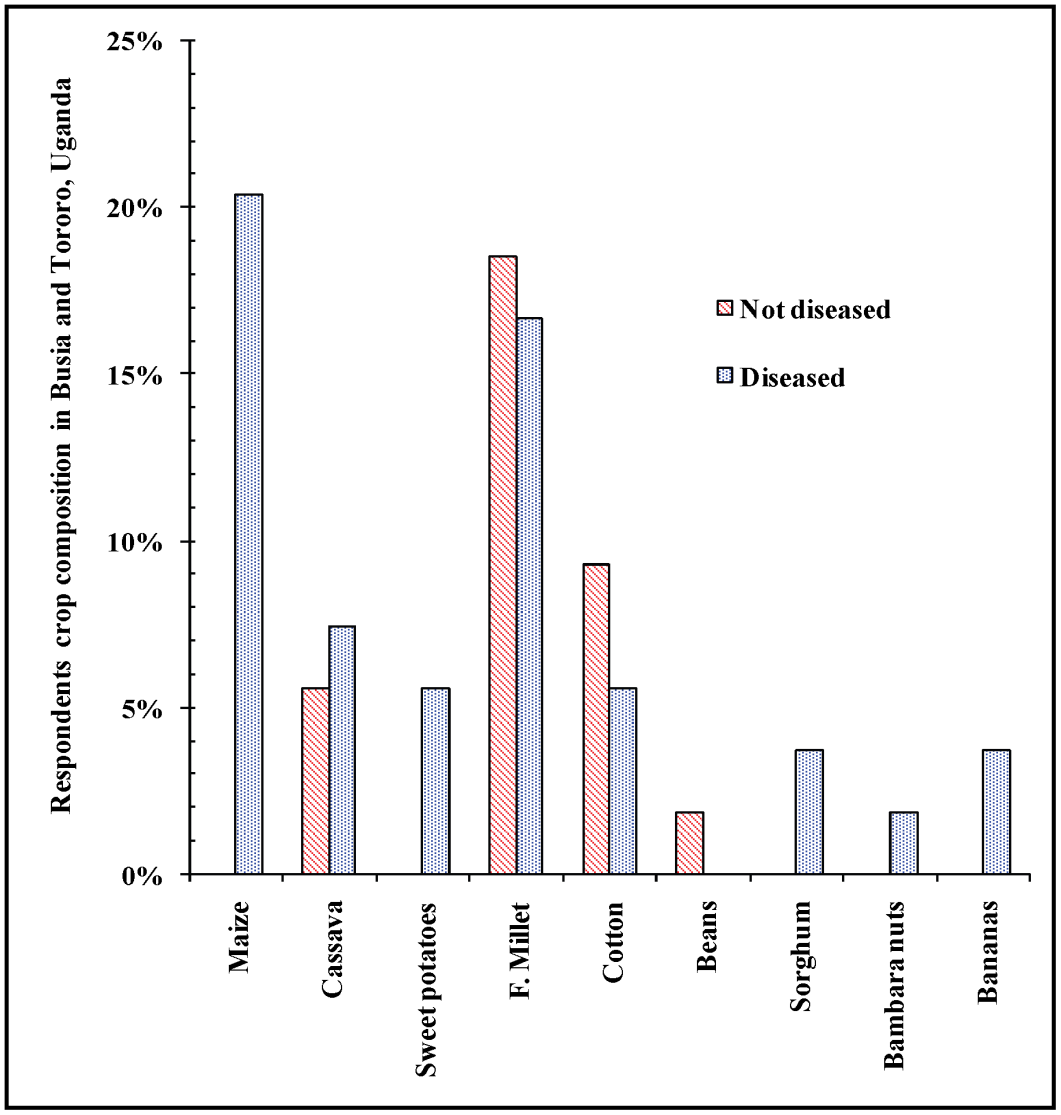
Crops grown in Southeast Uganda in 1970s in HAT affected and HAT free villages.

In Tororo (Ug) and Busia (Ug) Districts, Southeast Uganda, most respondents in both HAT affected villages and unaffected villages owned land from 1–5 acres (72.2%) and 6 to 10 acres (14.0%). At Southeast Uganda, the mean land size that each household occupied was 2.19 acres. Majority of respondents grow maize (37.1%), cassava (19.0%), finger millet (21.0%) and sweet potatoes (6.7%) (Table 5). Maize and cassava husbandry has been increasing from 1970s to date. Perennial crops such as cassava and bananas which can act as peri-domestic tsetse habitats were grown by 20.9% of the respondents in the year 2006. Cotton farming accompanied by intensive use of agrochemicals was practiced in 1970s and 1980 but declined in 1990s to late 2000s due to unsuccessful market performance and withdrawal of agriculture support policies. Cotton growing in unaffected villages was higher compared to HAT affected villages. The rest of the crops were almost constant standing crops in the fields. Other crops grown were soya beans, beans, groundnuts, sesame, vegetables and fruits such as oranges and pineapples. In 1970s crop farming (Figure 4) significantly differed (F [41]  = 17.490; p<0.05) between with HAT affected villages and unaffected villages in Tororo and Busia Districts, Southeast Uganda. HAT affected villages had more types of crops including cassava and bananas cultivated in the respondents' fields than unaffected villages which reported more millet, beans and crops using pesticides such as cotton.

### Livestock Keeping and Grazing Practices

Eighty percent (n = 608) of the total households in the selected villages in both countries owned livestock. The total livestock reared in the sampled study villages in Western Kenya were 1,416 and 2,781 in Southeast Uganda, respectively. Most respondents in Kenya (99.9%) and Uganda (67.3%) owned livestock albeit the total population of livestock in Uganda was twice that of Kenya. The villages recording higher cases of HAT had correspondingly higher number of animal and in general the cattle population has drastically reduced due to *nagana* (cattle trypanosomiasis) especially in Uganda. In Western Kenya the respondents mainly tethered their livestock (78.5%), free grazing (19.3%), practiced both tethering and zero grazing (0.7%) while 73.3%, 16.7% and 3.3% respectively were reported in Southeast Uganda. However, during the dry seasons cattle were driven to open grasslands along the rivers and in the swampy area. The communities in the study area sheltered their animals in close proximity to their household. Most goats and sheep were sheltered within the family households such as their kitchens.

### Drug Use in Livestock

Sixty five percent of the respondents in Western Kenya and 35% in Southeast Uganda generally treated their livestock. The most commonly used trypanocides was Veriben (33.0%), and the rest were Noviduim, Berenil and Samorin at low percentage of (0.9%) each and the rest did not treat their livestock. The low numbers in application of chemotherapy and chemoprophylaxis was due to fact that most trypanosomiasis drugs were expensive for the farmers to afford. Respondents in Western Kenya frequently used these drugs (72.7%) compared to Southeast Uganda (27.3%) citing drug cost as the impediment factor and also knowledge on the benefits of the treatment to human health. It was noted that other diseases such as tick-borne diseases and worms strongly constrained livestock husbandry at the Kenya and Uganda transboundary.

### Livestock Watering and Tsetse Interactions

**Figure 5:**
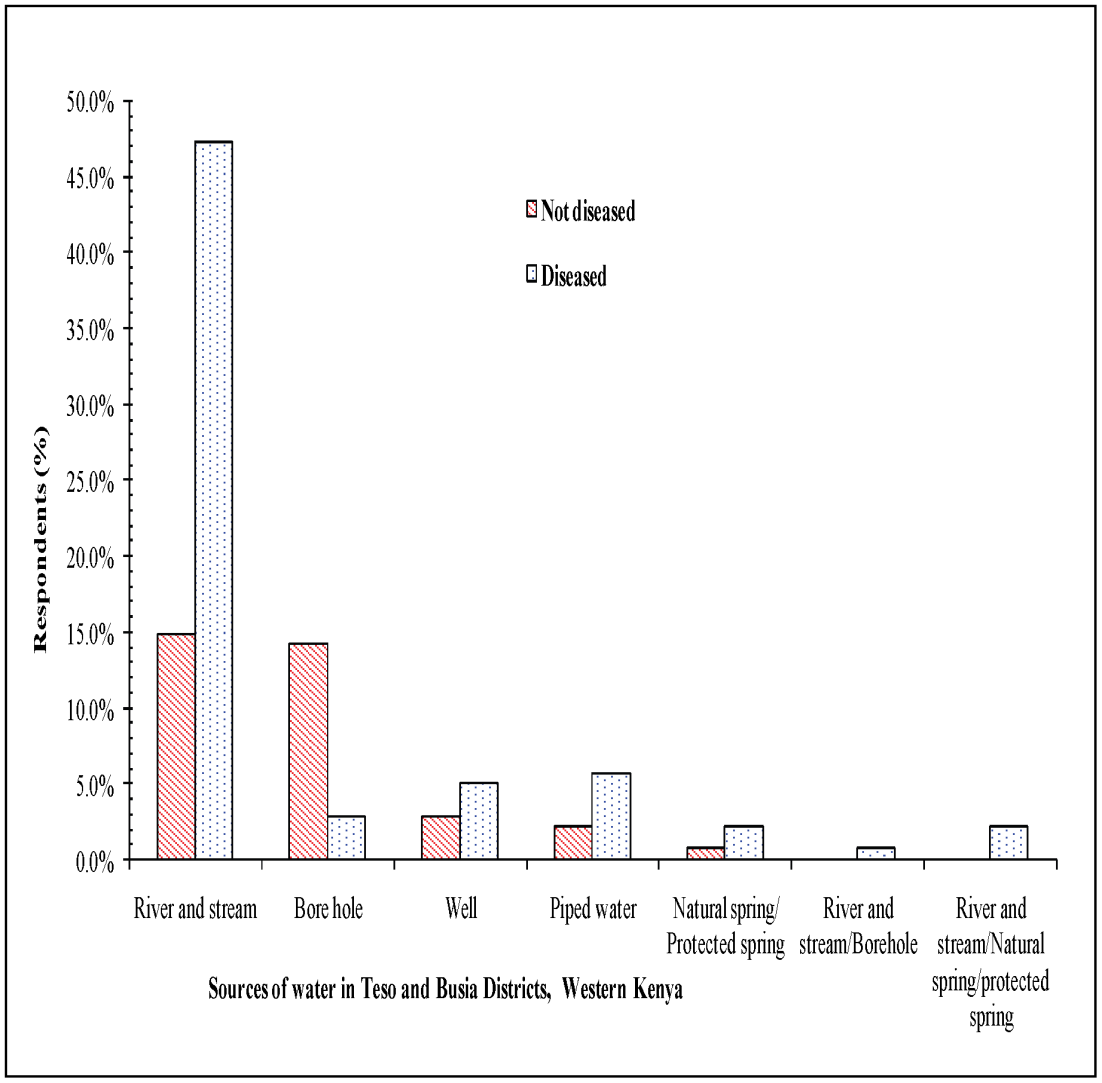
Sources of water in Western Kenya.

The respondents mainly watered their livestock from twelve noon to three o'clock in the evening (90.2%) and from nine to eleven o'clock (6.1%) (fig. 5). Five major watering livestock methods were identified namely, rivers and streams, boreholes, wells, piped water, protected spring and fetched water from rivers. In Kenya, the rivers or stream was the most preferred while in Uganda the boreholes and wells were better preferred. At the HAT affected villages it was found that threefold more respondent fetched river water compared to unaffected villages. It was also observed that wildlife, livestock and human shared watering points in both countries.

### Livestock Marketing

Livestock markets were not well established in Southeast Uganda compared to Western Kenya. In Busia (Ug) and Tororo (Ug) Districts in Southeast Uganda, farmers bought livestock from far markets and kept them in their homesteads for local re-selling within their homesteads. The major livestock markets for the respondents (32.7%, n = 251) in both countries were Funyula, Amukura (21.1%), Angurai (13.7%) in Kenya. The other markets were Pasindi (4.5%), Busaba (3.5%), Akapa (3.0%), Busolwe (2.5%) and the rest of the markets were visited by less than 2% of the respondents. Residents in the study area bought livestock from either country. In addition, some few respondents in both countries traveled to other districts such as Kumi (3.6%), Soroti Districts (1.8%) in Uganda and Bungoma District (0.5%) in Kenya to purchase livestock.

## Discussion

The outcome of WHO's committee on the social determinants of health has given an impetus to the consideration of social factors as crucial determinants of diseases [7], [42]. HAT, a neglected tropical disease presents special technological-based intervention challenges which are complicated with intricate socio-cultural issues. Basically, the transmission of HAT requires interaction of the human host, trypanosome, animal reservoirs, tsetse vectors, abiotic, and biotic environment. The major tribe of the respondents in Teso (Ke) and Busia (Ke) Districts study villages was Ateso (50.3%), followed by the Abaluhya (47.3%) tribe, other tribes such as were low (0.4%) as indicated in the results. In Busia (Ug) and Tororo (Ug) Districts selected villages main tribes were Abaluhya (59.8%), Adhola (Luo) (17.8%) and Ateso (13.7%).

A number of factors combine to explain the higher occurrence of HAT in Uganda than Kenya. Kenya had more respondents with primary, secondary and tertiary level of education compared their Ugandan counterparts in the selected villages. There were proportionally a higher number of farmers in Southeast Uganda compared to Western Kenya study villages. The results from this cross-sectional study further showed that tsetse flies were recognized by majority of respondents as important vectors of HAT in the study districts. However, some respondents did not know the vectors that transmit HAT especially in Southeast Uganda, while others mentioned vectors not specifically related to HAT (e.g. mosquitoes). Basic knowledge and understanding of the main biology, habitat, hosts and control methods of the main tsetse vectors in Southeast Uganda (*Glossina fuscipes fuscipes)* and Western Kenya (*Glossina pallidipes*) is a prerequisite for sound management of the fly population.

In Western Kenya more respondents (61%) knew more about tsetse and trypanosomiasis control methods compared to residents of Busia and Tororo Districts', Southeast Uganda (39.2%) therefore vector and trypanosomiasis control may be more in Kenya than in Uganda. Compared to Uganda, Kenya has had more application of bush clearing and live bait technology interventions in addition to chemoprophylaxis and chemotherapeutic measures over the years as reported in this study. Previously, it has been shown that farmers exposed to trypanosomiasis messages significantly had higher knowledge than those in the control areas or those not exposed to the messages [41], [43]. Busia (Ke) and Teso (Ke) Districts in Busia County, Western Kenya have particularly had concerted efforts to increase farmers' awareness of trypanosomiasis through initiating community education by use of posters and drama [41].

The respondents reported that male adults (28.4%) were at higher risk of contracting the disease followed by female adults (22.9%), and the children had low tsetse interactions due to their daily activities which corresponded with the actual analyzed gender HAT risk as was documented in this study finding. Herding livestock contributed the highest risk to tsetse interaction reporting 51.8% and 31.1% in Teso and Busia Districts in Busia County, Western Kenya and Busia and Tororo Districts, Southeast Uganda respectively. Social-cultural activities including bathing at the rivers and stream, fetching firewood and fishing; and occurrence of conducive woody vegetation especially in Uganda were important contributing factors to HAT occurrence in consonant with previous studies [10], [37]–[39], [41], [44].

In Teso and Busia Districts in Busia County, Western Kenya and Busia and Tororo Districts, Southeast Uganda, the most risky cultural activities were cleansing rituals contributing 45.5% and 58.0% respectively. Southeast Uganda compared to Western Kenya had higher HAT risk due to circumcision explained by the different ethnic composition of the study area in which circumcision rite was practiced by the Luhia communities such as Bagisu unlike in Kenya where they have been assimilated by the neighboring communities, therefore not practicing the rite with the exception of the Luhia Bukusu sub-tribe. The males, who engaged more in circumcision and cleansing rituals, and herding of animals which are performed in tsetse conducive habitats and for long durations, were more at risk in concord with known fact about the influence of human behavior on the epidemiology of a number of pathogens [39], [45]. Children had lower risks for the same reason. Farming and herding were perceived as the most risk occupation while ritual cleansing in agreement with Kokwaro *et al* [10] and circumcision ranked highly as risk factors of HAT acquisition. Although male circumcision was not practiced majority of residents (Luo, Adhola, Teso and Samia tribes) respondents perceived it as a cause of increased HAT risk.

Land use and land cover has immense influence on the occurrence of the tsetse flies and wildlife carriers of trypanosomiasis [29], [30]. The reason for this difference in LULC cover in a region of similar climatic zone can be explained by the past differences in management of politics, economies and tsetse and trypanosomiasis control efforts of the two states.

The density of vector population and proportion of the infectious vector determines the vector load and therefore the potential *per capita* risk burden on host [46]. The type of agriculture especially application of pesticides have strongly influenced HAT occurrence in the study area [41], [47]–[49]. Notably, cotton and tobacco farming areas had lower reported cases of HAT during the peak periods of the crops' farming (1970's), a trend that changed when, especially, cotton was abandoned and the farms invaded by *Lantana camara* bushes [50]. This coincided with HAT epidemic from 1976 to the early 1990s in Uganda. However, the presence of cassava as a crop type around the homestead has been significantly associated with HAT [39]. According to reports in 2002 Busia (Ug) District in Uganda had 37.9% and 55.5% of households growing maize and cassava, respectively [51].

During the dry seasons cattle are driven along the rivers and in the swampy area to graze which may increase the risk of livestock trypanosomiasis and human HAT due the close human-livestock-tsetse contact. Southeast Uganda also reported high livestock numbers compared to Western Kenya hence more HAT risk in the latter [6] explained by more tsetse fly catches in the traps [33], [34]. This has also been further supported by studies that demonstrate a positive spatial correlation of total cattle population and HAT occurrence in Western Kenya [6]. Zymodemes from domestic animals such as the cow and the pig have been found to be identical to those in man [52]. Pigs and cattle in the study area are known carriers of *Trypanosoma brucei brucei* and human infective *T.b.r* and the infection in domestic animals was not necessarily associated with signs of disease [31], [32], [35], [53]–[55]. Application of chemotherapeutic and chemoprophylaxis was hampered by the costs of drug and was reported to be low at 6.7% and 2.1% for Kenya and Uganda, respectively. Pest control targeting another important disease vector, the tick, has concomitantly controlled the HAT vectors. Successful installation of public dips and later increased private initiatives to install dips or spray livestock with acaricides ranging from dichlorodiphenyltrichloroethane (DDT), dieldrin and cypermethrin to organophosphates to amitraz in both countries in the 1980s and 1990 saw corresponding reduction in tsetse population.

Water sources for both livestock and domestic use may influence interaction among vectors and hosts. Other studies have reported that watering sites as major transmission foci of HAT [56]. Bathing or drawing water in the rivers and streams influenced HAT acquisition due to increased vector-human-wildlife-livestock interactions as reported by the respondents. If the tsetse fly bites an infected wildlife or domestic livestock HAT reservoir, it will transmit the disease to human beings during vector-human-wildlife –domestic animals interactions at water points. However, more studies needs to be carried out at the local scale at the transboundary to determine the impacts of watering management practices on HAT infection.

Livestock mobility has escalated in volume and speed between and within countries as the human population increased. Long distance trade of livestock facilitates the geographic redistribution of disease hosts and pathogens especially in Busia (Ug) and Tororo (Ug) Districts, Southeast Uganda where few markets for selling livestock existed than in Busia (Ke) and Teso (Ke) Districts in Busia County, Western Kenya with many established livestock markets. Cattle husbandry practices and marketing may affect the spread of HAT disease as reported in the study findings which showed that cattle increased HAT prevalence. Also the livestock congregation in markets (Kenya) and homestead (Uganda) may lead to more dissemination than isolated or less connected foci as farmers hold infected animals hosting human infective parasites. Occasional sale of animals across the boundary of the countries may lead transfer of HAT reservoirs between the countries, a possible explanation for the isolated case of HAT in 2007 in Kenya at an area that lacked conducive environment for tsetse fly.

During conflicts or difficult socio-economic and political times, vector control, active screening and treatment of potential HAT livestock reservoirs in order to eliminate circulating parasites are not undertaken [57]. Also farming activities which removes tsetse habitats has not been intensified in Uganda (29.4%) due to the civil unrest compared to Kenya (58.2%) [44].

In Western Kenya there have been continuous efforts to eradicate tsetse in the past three decades. Kenya has had a well-established socio-economic unit at KARI-TRC (formerly KETRI) which interacts with the communities in order to create awareness and transfer of knowledge and technology aimed at controlling HAT. At KARI-TRC the research teams apply the multipronged approach for tsetse and trypanosomiasis control where biological, economic, socio-cultural and environmental issues are considered in addition to engaging community participation. HAT has consequently been reduced through the targeted multi-disciplinary approach by KETRI, Kenya government, relevant stakeholders and donors. It is no longer considered a significant public health problem. However in Southeast Uganda, *T. b. rhodesiense* epidemics have been associated with civil strife [5] when collapse of essential services such as health, veterinary and vector control have occurred. In the past decade the concerted efforts of the Ugandan government and donor programs such as Farming In Tsetse Controlled Areas (FITCA) program, Pan African Tsetse and Trypanosomiasis Eradication Campaign (PATTEC) in conjunction with other stakeholders in old HAT endemic foci reduced HAT cases to minimal numbers. However, HAT has spread to virgin foci away from the traditional areas in Southeast Uganda.

This study found that the differences in the social behavior of the communities who are similar in many respects but have experienced differences in national socio-politics influenced tsetse and trypanosomiasis control policy formulation and action. This explained the higher number of HAT cases in Uganda than Kenya. Otherwise, the communities across the borders are similar with regards to cultural practices, climatic and environmental living conditions, income, and literacy (measured as acquisition of basic education). HAT occurrence was found to be directly related to contacts between the study population and tsetse habitats so that socio-economic and cultural activities which entailed closer proximity to the tsetse enhanced the disease (Table 1) [10]. This study also demonstrated that increased cattle and pigs enhanced HAT occurrence as indicated by other studies. In addition lack of established livestock markets in, Southeast Uganda where cattle traders held their commodities (mostly untreated) within their homesteads for long periods before being re-sold exacerbated HAT risks compared to Kenya. Hence this study encourages multi-stakeholder participation in tsetse and trypanosomiasis control of both concerned communities, ministries of livestock and health to effectively manage HAT.

Continuous and systematic approach to tsetse and trypanosomiasis control eliminates HAT as demonstrated by Kenya. Kenya has had much less occurrence of HAT through the application of simple methods such as habitat destruction, treatment and live bait technology using synthetic pyrethroids reduced or eliminated tsetse, circulating parasites in both the vectors and animal carriers.

We conclude that control and prevention of HAT disease requires adequate knowledge of interactions among factors such as human behavior, the environment, and the life cycles of parasites. Therefore, there is need to educate communities to bring changes on their belief, socio-economic and cultural practices to protect themselves from tsetse bites using available effective and long term sustainable tsetse and trypanosomiasis control methods. There is need for national health services to include tsetse and trypanosomiasis programs for systematic and continuous control efforts targeting the vector and the parasites causing disease.

This study recommends that a place with similar culture, tribe, climate, physical features, historical can have considerable difference in epidemiology of disease that was attributed to the socio-economic and political differences. Therefore socio-political and economic environment play a big in the management of neglected tropical diseases.

## Supporting Information

Checklist S1 **Formatted STROBE checklist.**
(DOC)
